# Effect of Low-Temperature Preservation in Optisol-GS on Preloaded, Endothelium-Out DMEK Grafts

**DOI:** 10.3390/jcm12031026

**Published:** 2023-01-28

**Authors:** Alessandro Ruzza, Stefano Ferrari, Matteo Airaldi, Vito Romano, Diego Ponzin

**Affiliations:** 1 Fondazione Banca degli Occhi del Veneto, 30174 Venice, Italy; 2 Department of Medical and Surgical Specialties, Radiological Sciences, and Public Health, Ophthalmology Clinic, University of Brescia, 25121 Brescia, Italy; 3 Eye and Vision Science, Institute of Life Course and Medical Sciences, University of Liverpool, Liverpool L3 5TR, UK

**Keywords:** DMEK, eye bank, preloaded, temperature

## Abstract

The aim of the study was to assess different temperature ranges for the preservation of pre-loaded Descemet Membrane Endothelial Keratoplasty (DMEK) grafts in the DMEK RAPID Mini device. Methods: Three groups of 15 DMEK grafts (five per group) were pre-loaded in the DMEK RAPID Mini and preserved in Optisol-GS for 72 h at different temperatures: group A at >8 °C, group B between 2–8 °C and group C at <2 °C. After stripping and preservation, the viability of the endothelium, cell loss and morphology were assessed through light microscopy following trypan blue and alizarin red staining. Results: Overall mortality was 4.07%, 3.97% and 7.66%, in groups A, B and C, respectively, with percentages of uncovered areas of 0.31%, 1.36% and 0.20% (all *p* > 0.05). Endothelial cell density variation was 5.51%, 3.06% and 2.82% in groups A, B and C, respectively (*p* = 0.19). Total Endothelial Cell Loss (ECL) was 4.37%, 5.32% and 7.84% in groups A, B and C, respectively (*p* = 0.39). Endothelial cell morphology was comparable in all three groups. Conclusions: In the DMEK RAPID Mini, low temperatures (<2 °C) may affect the quality of pre-loaded grafts, inducing a higher ECL after 72 h of preservation, although no significant differences among groups could be proved. Our data would suggest maintaining grafts loaded in the DMEK RAPID Mini at temperatures between 2–8 °C for appropriate preservation.

## 1. Introduction

Eye banks are playing a crucial role in the development of more standardized and surgeon-friendly techniques for preparing, loading, transporting and delivering grafts for Descemet Membrane Endothelial Keratoplasty (DMEK) [1,2,3,4]. In the early years of the development of this technique, surgeons had to prepare the grafts autonomously just before transplantation, increasing surgical time and exposing themselves to the risk of graft preparation failure. Owing to the experience gained by eye banks in the preparation of grafts for Descemet Stripping Automated Endothelial Keratoplasty, the separation of the Descemet Membrane (DM) from the stroma, the trephination at the desired diameter, and loading of the graft into the injector are better off carried out by technicians routinely involved in such preparations [5,6,7].

In the eye bank, the DM can be loaded inside the injector either with the corneal endothelium folded outwards (ENDO-OUT configuration) or inwards (ENDO-IN configuration). Once separated completely from the posterior stroma, the DM naturally adopts a scrolled, ENDO-OUT configuration, an effect likely due to the different degrees of elasticity of the anterior and posterior surfaces of the DM [8]. Instead, in the ENDO-IN configuration, the membrane is folded in the opposite way to its natural scrolling tendency. Folding can take several minutes to be carried out, but once inside the patient’s eye, it requires minimal time for unfolding, since it is perceived as an unnatural conformation [9]. Despite such differences, both techniques have been reported to lead to comparable results in terms of visual outcomes and it is up to the surgeon’s expertise and skill to decide which one to use for DMEK [10,11].

Eye banks have been collaborating with surgeons to provide pre-trephined and pre-loaded membranes preserved either with ENDO-IN or ENDO-OUT configurations. To improve such preparations, efforts are being made to minimize Endothelial Cell Loss (ECL) associated with preparation [2,3,12,13], loading [11,14,15,16], preservation and transportation [1,17,18,19,20,21] and, finally, injection [10,11,14,15,22] of DMEK grafts.

While no dedicated device is so far available for membranes folded inwards, the DMEK RAPID Mini system (Geuder AG, Heidelberg, Germany) has recently received CE marking for the transportation of pre-loaded, ENDO-OUT grafts [17]. This system was initially designed for corneal tissues preserved in organ culture (OC) and stored at intervals ranging from room temperature to 31 °C. However, the majority of eye banks outside Europe use cold storage (CS) as the preferred method for preservation, with corneal tissues placed in single small vials and kept in a refrigerator at 2–8 °C. The DMEK RAPID Mini has been recently adapted for CS. The idea behind the CS of DMEK grafts is that cold temperature reduces cellular demand for metabolic energy [23,24]. However, concerns regarding the shorter conservation time and possibly higher endothelial cell mortality associated with hypothermia exist [24,25,26], highlighting the need for a deeper understanding of the effects of different low-temperature ranges on endothelial viability in preloaded DMEK grafts.

The aim of this study was, therefore, to evaluate the effects of preservation at different low-temperature ranges on corneal endothelial cells viability, simulating the transport of pre-loaded DMEK grafts in the DMEK RAPID Mini device.

## 2. Materials and Methods

Donor corneal tissues unsuitable for transplantation due to poor endothelial cell counts, procured by Fondazione Banca degli Occhi del Veneto Onlus (Venice, Italy) were used for the research purposes and the validation studies described in this manuscript, according to the realms of the law 91/99 and after an informed consent form was signed by the donor’s next of kin.

### 2.1. Tissue Preparation

In this study, we used fifteen corneas (*n* = 15) retrieved from the morgue and preserved in Cold X Medium (homemade medium) at 4 °C for up to 3 days. The membranes were prepared following a standard stripping protocol as previously reported [2], using a 9.5 mm diameter punch (Moria, Antony, France) and leaving a small area of peripheral adhesion between DM and stroma. After the stripping step, all tissues were stained with 0.25% Trypan Blue dye (Gibco, NY, USA) and evaluated by light microscopy (Axiovert, Zeiss, Oberkochen, Germany). If deemed acceptable, the grafts were punched again with an 8.25 mm punch and completely detached from the stroma. The corneal concavity was completely filled with Optisol-GS (Rochester, NY, USA). Having enough space, the membranes roll up on themselves assuming a cylindrical shape and exposing the endothelium outwards. The membranes were then loaded into the DMEK RAPID Mini device as previously described [17]. Briefly, the DMEK RAPID Mini device consists of a front plug connected to the tip of an injector from one side and to a silicone tube on the other. A 5 mL syringe is attached to the silicone tube connector. The whole system must be filled with Optisol-GS, to avoid any air bubble formation. The folded membrane is sucked into the device by means of a syringe vacuum through the posterior part of the injector. The back of the injector is then blocked using the rear plug. In this study, the whole system was fixed in the transportation holder and finally placed in the dedicated vial filled with Optisol-GS.

The tissues were divided into three groups (A, B and C) and stored at different temperatures, as described below:

- Group A: >8 °C storage (*n* = 5);
- Group B: 2–8 °C storage in a “transport simulation” condition (*n* = 5);
- Group C: <2 °C storage (*n* = 5).

Transport simulation was performed by leaving the pre-loaded tissues on a laboratory shaker (Duomax 1030, Heidolph, Schwabach, Germany) placed inside a temperature-monitored refrigerator for the entire duration of the experiment. All the membranes were preserved for 72 h.

### 2.2. Tissue Evaluation and Study Outcomes

The tissues were analyzed after the stripping step (T0). The membranes were stained with trypan blue dye and evaluated by means of light microscopy (Primovert; Zeiss, Jena, Germany) using a hypotonic analysis solution (HAS) to highlight the intercellular borders and to assess Endothelial Cell Density (ECD), cell mortality rate, acellular areas (extensive blue-stained portions on the membrane with no traces of endothelial cells). To be included in this study, tissues had to have an ECD > 1700 cells/mm^2^, a mortality rate lower than 2.5% and percentages of uncovered areas lower than 1% at T0.

After 72 h of preservation (T72), the membranes were carefully extracted from the injector, gently placed and unfolded on a glass slide, stained with trypan blue dye and immersed in hypotonic analysis solution to perform the second light microscopy evaluation.

**Figure 1:**
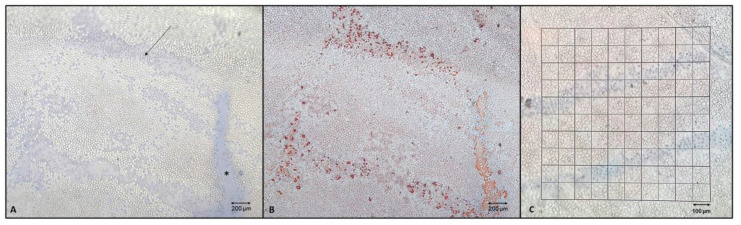
(**A**) Group B, 40× magnification, Trypan Blue and Alizarin Red, after 72 h of preservation. Cornea with trypan blue positive cells (arrow) on folds and de-cellularized areas (asterisk) displayed by dye suffusion onto Descemet’s membrane. (**B**) Same tissue but stained by Alizarin red dye which only stains the Descemet’s membrane, clearly showing the cell margins and the uncovered areas, confirming what observed with the Trypan Blue staining. (**C**) 100× magnification, Trypan Blue, archive photo. Trypan blue positive cells localized on the Descemet folds. To perform the mortality assessment, the reticle is overlayed to the image and then the number of small squares covering the areas are counted. To get the percentage, this value is referred to the whole surface of the membrane.

In general, trypan blue positive cells appear within the corneal endothelium as a result of different possible discomfort events or situations, such as poor initial quality of the tissue, long post-mortem time, long storage time, stressful storage conditions, iatrogenic trauma, etc. All these conditions lead to changes in the cell membrane permeability, allowing the dye to stain the cellular nuclei. These cells are considered dead and ready to detach from the Descemet membrane. Uncovered (or acellular) areas are a natural consequence of cellular deaths or are caused by severe mechanical traumas leading to cell detachment (Figure 1).

To estimate the ECD and the extent of damaged areas, the cells in five 1 mm^2^ squares of a 10 × 10 mm reticule inserted in the eyepiece of an inverted microscope were counted manually at 100× magnification. The number of trypan blue positive cells allowed us to determine the percentage of cell death and uncovered areas (Figure 1) in relation to membrane surface diameters of 9.5 mm (70.85 mm^2^) and 8.25 mm (53.43 mm^2^). For the confirmation of uncovered areas, Alizarin red (5%; ThermoFisher Scientific, Waltham, MA, USA) was topically applied, and the tissues were incubated for 5 min at RT away from bright light. The tissues were then re-evaluated under the light microscope.

The overall mortality was obtained by calculating the difference between the mortality detected at T72 (post-preservation) and the mortality found at T0 (post-stripping). The overall uncovered areas were calculated using the same method. By doing so, the damage that occurred during the stripping phase could be excluded from the final data evaluation, which will be only referred to in the conservation phase. Total ECL was obtained by combining these two values (overall uncovered areas plus overall mortality).

### 2.3. Statistical Analysis

Descriptive data were summarized using the mean (standard deviation) and percentages where appropriate.

The normal distribution of outcomes was checked by means of Shapiro–Wilk tests and visual inspection of Q-Q plots.

One-way ANOVA was used to compare the effect of the 3 temperature groups on ECD, mortality rate and uncovered areas at T0 and at T72, as well as ECD variation, overall mortality, overall uncovered area and total ECL between T0 and T72. Homogeneity of variance and covariance was confirmed by means of Levene’s test and Box’s M-test, respectively.

Post hoc pairwise *t*-tests with Bonferroni correction were used for comparisons between groups in case of a significant ANOVA result.

A *p*-value of 0.05 was considered statistically significant. All analyses were conducted using R software version 4.2.2 (R Project for Statistical Computing, Vienna, Austria; R Foundation for Statistical Computing; 2022, https://cran.rproject.org accessed on 3 December 2022).

## 3. Results

Membranes with similar biological features were used for all the groups (Table 1).

After stripping (T0), in Group A the mean ± SD ECD was 2160 ± 134 cells/mm^2^, the mortality rate was 0.26 ± 0.31% and the percentage of uncovered areas was of 0.16 ± 0.27%. In Group B, the average ECD was 2110 ± 260 cells/mm^2^, the mortality rate was 1.02 ± 0.21% and the percentage of uncovered areas was of 0.07 ± 0.16%. In Group C, the average ECD was 2060 ± 134 cells/mm^2^, the mortality rate was 0.54 ± 0.47% and the percentage of uncovered areas was of 0.14 ± 0.28% (Table 2).

At T0, only the mortality rate was significantly different among the three groups (ANOVA, *p* = 0.01). The post hoc pairwise comparison showed that the mortality rate was significantly greater in group B compared to group A (*p* = 0.01).

The average storage temperature recorded by the probes in the refrigerator was 9.3 °C in group A, 5.1 °C in group B and 1.3 °C in group C. After preservation (T72), in Group A the mean ± SD ECD we found was 2040 ± 114 cells/mm^2^, mortality was 4.33 ± 3.88% and the percentage of uncovered areas was of 0.34 ± 0.50%. In Group B, the ECD was 2044 ± 246 cells/mm^2^, mortality was 4.99 ± 2.12% and the percentage of uncovered areas was 1.43 ± 1.42%. In Group C, the ECD was 2000 ± 100 cells/mm^2^, mortality was 8.19 ± 4.93% and the percentage of uncovered areas was 0.32 ± 0.36%.

At T72, no significant difference in ECD, mortality rate or uncovered areas could be identified (all *p* > 0.05).

The mean ± SD ECD variation between T0 and T72 was 5.51 ± 1.79%, 3.06 ± 2.80% and 2.82 ± 2.58% in Group A, B and C, respectively (*p* = 0.19). Likewise, no difference was found in Overall Uncovered Areas (*p* = 0.1). In group C, Overall Mortality (7.66 ± 4.95%) and Total ECL (7.84 ± 4.94%) were higher than in group A (4.07 ± 3.76% and 4.37 ± 3.86%) and group B (3.97 ± 1.97% and 5.32 ± 2.90%). However, these differences were not statistically significant (*p* = 0.25 and *p* = 0.39).

**Figure 2:**
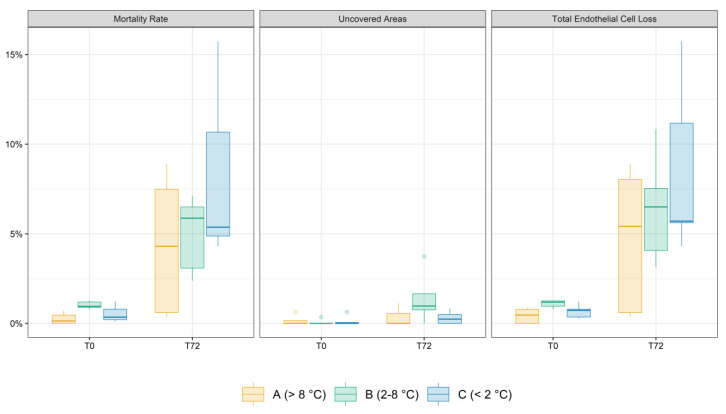
Boxplots displaying the distribution of mortality rate, uncovered areas and total Endothelial Cell Loss (ECL) in the three groups at T0 and T72. The solid lines represent the median, the boxes span the 25th and 75th quantile, the whiskers of the boxes extend to 1.5 times the interquartile range, and solid dots represent outliers. Only mortality rate was significantly different among the three groups at T0, and post hoc pairwise comparison showed that it was significantly greater in group B compared to group A (*p* = 0.01). At T72, mortality rate and total ECL showed a trend for inversely proportional correlation to temperature, although these differences did not reach statistical significance.

Boxplot graphical representations of the distribution of mortality rate, uncovered areas and total ECL at T0 and T72 in the three groups are shown in Figure 2.

### Light Microscopy Samples

**Figure 3:**
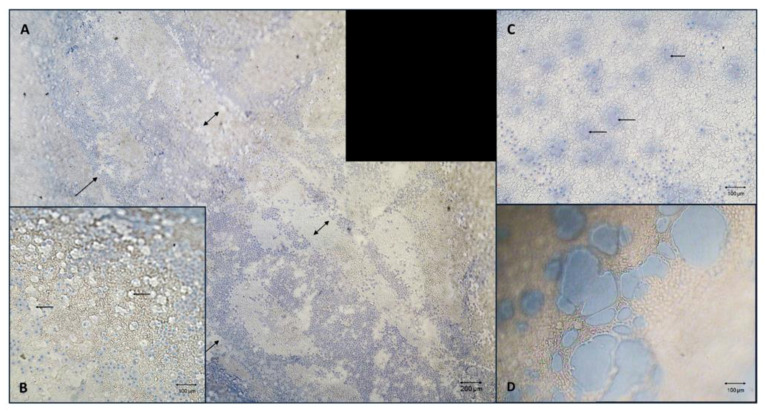
(**A**) Group C, 40× magnification, Trypan Blue/V9, after 72 h of storage, blending of two images. Long area (approximately 5 mm) of localized mortality with well-defined margins (arrows) located in the middle periphery of the membrane. This shape of mortality was also observed on membranes stored in other groups. (**B**,**C**) Group C, 100× magnification, Trypan Blue/V9, after 72 h of storage. Diffuse presence of Trypan Blue positive cells and uncovered areas (arrows) homogeneously distributed throughout the Descemet membrane endothelial surface. (**D**) 100× magnification, Trypan Blue/sucrose, preserved at 4 °C, archive photo (2010). After being retrieved from the morgue, the tissue was preserved in a vial with tissue culture medium and mistakenly put in direct contact with an ice pocket. After 12 h of preservation, the endothelium showed circular areas with absence of cells and a high percentage of scattered mortality throughout the endothelial surface.

In all groups, well-defined areas of trypan blue positive cells (Figure 3A) located in different parts of the membrane and especially in the mid-periphery, could be identified. These linear stress marks could be attributable to friction, stripping and/or handling of the grafts. However, three tissues in Group C displayed scattered mortality with the presence of small acellularized areas (Figure 3B,C), likely due to widespread cellular sufferance caused by the low storage temperature conditions (Figure 3D).

## 4. Discussion

In this study, DMs were exposed to three different temperature ranges to verify the best conditions for cell survival during preservation in Optisol-GS within a DMEK RAPID Mini device in the endothelium folded outwards configuration. At the end of the experiment, a higher mortality and higher total ECL were detected in Group C (preservation at <2 °C) compared to that seen in Group B (2–8 °C) and A (>8 °C), although these differences did not reach statistical significance.

Along with preservation time, the temperature is a crucial parameter for appropriate corneal tissue preservation, especially when the isolated Descemet Membrane, and its endothelium, are preserved [24]. The findings of this study seem to confirm that storage temperature plays an important role in maintaining endothelial cell viability [23]. Low temperatures (<2 °C) can disrupt the cellular junctions leading to plasmatic membrane breakage and, consequently, increase the cell mortality rate [27,28]. Low storage temperatures have also been shown to affect cellular metabolism [25,26], thus reducing the endothelium’s ability to reorganize and replenish the exposed areas as a consequence of cellular death. Conversely, a rearrangement of the endothelial mosaic can be observed following a metabolic activation triggered by storage at higher temperatures, as shown in previous studies on epithelial cells [29,30]. This could explain why a progressive ECD variation was found in tissues preserved at different temperatures, such as in Group A compared to Group C, an effect likely due to endothelial repair reactivation caused by increased temperatures [31]. Finally, studies on cultured endothelial cells show that the oxidative stress generated by low storage temperatures might be particularly damaging to the mitochondria-rich endothelial cell [24,32]. Mitochondria are, in fact, abundant in the highly interdigitated apical complexes of endothelial cells, indicative of their crucial role in active fluid transport and cellular adhesion [33]. Cold storage conditions paradoxically enhance the formation of reactive oxygen species, promoting adhesion loss and non-apoptotic cell death of mitochondria-rich endothelial cells [32,33,34].

To date, two main storage systems of corneal tissue have been developed: cold storage, mainly used in America and Asia, maintains the cornea between 2 °C and 8 °C for a maximum of 14 days, while Organ Culture preservation, which uses room temperature up to 31–37 °C for storage of corneas, can preserve the tissues for up to 28 days [1,18,23]. The advantages of cold storage (no microbiology, no serum, less cost and storing space, ease of transport) must be weighed against those of organ culture, which is more technically difficult and time-consuming but can support cell metabolism under physiologic conditions and maintain cell viability for longer periods of time [20,23,24,30,31].

In this study, the preservation of corneal tissue was performed in Optisol-GS, the most widely employed medium for CS [23]. Other studies have employed organ culture medium for the storage and analysis of preloaded DMEK grafts stored in the DMEK RAPID system, with results comparable to ours. In particular, Wojcik and colleagues found a total ECL of 3.3–11.5% after storage and transport at room temperature in the DMEK RAPID device with tissue culture medium (TCM) supplemented with Dextran, a commonly used deswelling agent [17]. Català and colleagues found a total ECL of 16.7% and uncovered areas of 8% after transportation at an ambient temperature of endothelium-out DMEK grafts in the DMKE RAPID device employing analogous TCM and 6% Dextran [18].

As DMEK continues to evolve, optimization of the preparatory steps leading to surgery will represent more and more a key area for development. The efforts to minimize ECL in the preparation, storing and shipping of preloaded grafts are paramount given its prominent role in early postoperative ECL. In fact, preoperative ECD has been directly correlated with 6-month post-DMEK ECD, which in turn was found to be a key predictor of late endothelial failure. Specifically, a 6-month ECD count of less than ~830 cells/mm^2^ has been associated with a significantly lower 5-year graft survival rate in a study by Vasiliauskaitė and colleagues [35]. Given the multifactorial, high variability in tolerance to the cellular stress caused by stripping and preservation [1,3], the authors suggest that identification and exclusion by Eye Banks of “low-performing” grafts may ensure that only grafts with good tolerance to manipulation and, therefore, with higher postoperative, ECD counts get transplanted [35].

Limitations of this study could be attributed to damage to the endothelium not related to hypothermic storage, which could have confounded our results. In the pre-loaded, ENDO-OUT configuration, the membrane is left free to roll up and then vacuumed through a tubing system connected to a syringe and can be accommodated in a larger space as compared to the devices for ENDO-IN membranes. Since the graft spontaneously rolls up with the endothelium folded outwards, corneal endothelial cells may be exposed to a certain degree of friction against the internal walls of the injector, especially during the injection phase when the diameter of the funnel is reduced [1]. Furthermore, some of the variability in the degree of overall mortality could also be influenced by the unpredictable folding of the DMs, spontaneously assuming configurations such as the double scroll shape [36,37], and consequently exposing different regions of the endothelium. However, we observed well-defined areas of trypan blue positive cells, located in different peripheral areas of the membrane, across all three different groups, meaning that the effects of friction, stripping and/or handling of the DMs have most likely equally affected all tissues in our study. Indeed, the degree of ECL is in line with previous studies using the DMEK RAPID system [17,18] or other alternative devices used for transporting preloaded and pre-stripped grafts for DMEK [15,21,38].

Finally, given the small sample size available, we might have been underpowered to detect significant differences among the three groups (Post Hoc Power range = 0.1–0.85).

## 5. Conclusions

In conclusion, to preserve the quality of isolated DMEK grafts folded in the ENDO-OUT configuration in the DMEK RAPID Mini device in Optisol-GS, it is recommended to keep the grafts at temperatures between 2 to 8 °C. Lower temperatures can alter the morphological characteristics of the endothelium and possibly increase cell mortality. Conversely, no major issue has been observed with higher temperatures as long as these are kept below room temperature to avoid fungal growth [39].

## Figures and Tables

**Table 1:** Post-stripping and post-preservation endothelial cell density, mortality rate and uncovered areas in Group A (storing for 72 h post-stripping in Optisol-GS at >8 °C), Group B (2–8 °C) and Group C (<2 °C).

		Post Stripping (T0)			Post Preservation (T72)		
N	Preservation	ECD (cells/mm^2^)	Mortality (%)	Uncovered Areas (%)	ECD (cells/mm^2^)	Mortality (%)	Uncovered Areas (%)
1	>8 °C	2100	0	0	2000	0.37	0
2	>8 °C	2100	0	0	2000	0.60	0
3	>8 °C	2300	0.14	0.64	2100	8.89	0
4	>8 °C	2000	0.47	0	1900	7.48	0.60
5	>8 °C	2300	0.71	0.16	2200	4.31	1.11
6	2–8 °C	1800	1.19	0	1800	7.10	3.74
7	2–8 °C	2300	1.27	0	2300	6.50	0
8	2–8 °C	2000	0.96	0	1900	2.38	0.76
9	2–8 °C	2000	0.78	0	1900	3.09	0.97
10	2–8 °C	2450	0.90	0.35	2320	5.87	1.66
11	<2 °C	2200	0.79	0	2100	10.67	0.50
12	<2 °C	2200	1.23	0	2100	5.37	0.24
13	<2 °C	2000	0.35	0	1900	15.74	0
14	<2 °C	2000	0.21	0.06	2000	4.31	0
15	<2 °C	1900	0.10	0.64	1900	4.87	0.83

ECD, Endothelial Cell Density; N, number.

**Table 2:** Average values and differences in Endothelial Cell Density, Mortality and Uncovered Areas at T0 and T72.

	Groups			
	A, *n* = 5 Mean (SD)	B, *n* = 5 Mean (SD)	C, *n* = 5 Mean (SD)	*p* *
Post-stripping (T0)				
ECD (cells/mm^2^)	2160 (134)	2110 (261)	2060 (134)	0.7
Mortality (%)	0.26 (0.31)	1.02 (0.21)	0.54 (0.47)	0.01
Pairwise comparison ^#^:				
Group A vs. Group B				0.01
Group A vs. Group C				0.23
Group B vs. Group C				0.09
Uncovered areas (%)	0.16 (0.27)	0.07 (0.16)	0.14 (0.28)	0.84
Post-extraction (T72)				
ECD (cells/mm^2^)	2040 (114)	2044 (246)	2000 (100)	0.9
Mortality (%)	4.33 (3.88)	4.99 (2.12)	8.19 (4.93)	0.27
Uncovered areas (%)	0.34 (0.50)	1.43 (1.42)	0.32 (0.36)	0.12
T0–T72 difference				
ECD Variation (%)	5.51 (1.79)	3.06 (2.80)	2.82 (2.58)	0.19
Overall Mortality (%)	4.07 (3.76)	3.97 (1.97)	7.66 (4.95)	0.25
Overall Uncovered areas (%)	0.31 (0.44)	1.36 (1.42)	0.20 (0.20)	0.1
Total Endothelial Cell Loss (%)	4.37 (3.86)	5.32 (2.90)	7.84 (4.94)	0.39

ECD, Endothelial Cell Density; *n*, number; SD, standard deviation. * One-way ANOVA. ^#^ Post hoc pairwise comparison with *t*-test using Bonferroni correction.

## Data Availability

Data is contained within the article.
